# PE11, a PE/PPE family protein of *Mycobacterium tuberculosis* is involved in cell wall remodeling and virulence

**DOI:** 10.1038/srep21624

**Published:** 2016-02-23

**Authors:** Parul Singh, Rameshwaram Nagender Rao, Jala Ram Chandra Reddy, RBN Prasad, Sandeep Kumar Kotturu, Sudip Ghosh, Sangita Mukhopadhyay

**Affiliations:** 1 Laboratory of Molecular Cell Biology, Centre for DNA Fingerprinting and Diagnostics (CDFD), Nampally, Hyderabad, India; 2 Graduate Studies, Manipal University, Manipal, Karnataka, India; 3 Centre for Lipid Research, CSIR-Indian Institute of Chemical Technology, Uppal Road, Hyderabad, India; 4 Molecular Biology Division, National Institute of Nutrition (ICMR), Jamai-Osmania PO, Hyderabad, India

## Abstract

The role of the unique proline-glutamic acid (PE)/proline-proline-glutamic acid (PPE) family of proteins in the pathophysiology and virulence of *Mycobacterium tuberculosis* is not clearly understood. One of the PE family proteins, PE11 (LipX or Rv1169c), specific to pathogenic mycobacteria is found to be over-expressed during infection of macrophages and in active TB patients. In this study, we report that *M. smegmatis* expressing PE11 (*Msmeg-PE11*) exhibited altered colony morphology and cell wall lipid composition leading to a marked increase in resistance against various environmental stressors and antibiotics. The cell envelope of *Msmeg-PE11* also had greater amount of glycolipids and polar lipids. *Msmeg-PE11* was found to have better survival rate in infected macrophages. Mice infected with *Msmeg-PE11* had higher bacterial load, showed exacerbated organ pathology and mortality. The liver and lung of *Msmeg-PE11-*infected mice also had higher levels of IL-10, IL-4 and TNF-α cytokines, indicating a potential role of this protein in mycobacterial virulence.

*Mycobacterium tuberculosis*, the causative agent for tuberculosis (TB) employs several strategies to modulate the host immune responses to favor its intracellular survival^1^. It possesses a unique proline-glutamic acid (PE)/proline-proline-glutamic acid (PPE) family of proteins whose role in the pathophysiology and virulence is not clearly understood^2^. The *M. tuberculosis* PE/PPE family has a conserved PE and PPE motif at the N-terminal domain with variations in the C-terminal region^2^ and is found to be highly expanded in the pathogenic strains of mycobacteria^3^. The PE and PPE proteins have been thought to play important roles in generating antigenic variation and immune evasion, some of them are known to induce strong B cell response^1^. However, their exact pathophysiological roles are yet to be fully understood. A detail functional study of these genes is, therefore, crucial to understand the host-pathogen interactions and survival strategies adopted by mycobacteria.

The unique and highly complex cell wall of mycobacteria is implicated for its resistance to various anti-mycobacterial drugs and environmental stressors^4^. The cell wall contains distinctive lipids and glycolipids that contribute to extreme hydrophobicity to the outer surface. These lipids which include mycolic acids, phosphatidyl inositol mannosides, phthiocerol dimycocerosates and lipoglycans such as lipomannan and lipoarabinomannan play important roles in maintaining integrity of the cell envelope^5^. The mycobacterial lipids are also involved in modulating early immune responses of macrophages to infection^6^ like resistance to free radicals, prevention of phagosomal maturation and inhibition of synthesis of anti-mycobacterial cytokines by the host immune system^7^. Thus, the cell wall lipids are critical to the mycobacterial virulence process^7^,^8^. Due to its increasing resistance to current chemotherapeutic agents, it is important to identify newer generation anti-mycobacterial drugs and the processes/components involved in the cell wall lipid synthesis present an attractive target for this purpose^9^. Interestingly, some of the currently available drugs like isoniazid^10^ and ethambutol^11^ are known to inhibit mycobacterial cell wall synthesis.

The *M. tuberculosis* lipases/esterases play crucial roles in lipid metabolism and mycobacterial physiology^12^. Lipases/esterases and phospholipases are molecules that provide metabolic turnover of lipids and can be defined as essential biocatalysts for the hydrolysis of esters containing long-chain and short-chain fatty acids. These fatty acids, on one side provide energy for intracellular persistence of the dormant bacilli and its reactivation and on the other side they can act as precursors for the cell wall lipids and are thought to contribute to virulence and pathogenicity of the bacilli^13^. *In silico* analysis of *M. tuberculosis* genome predicted presence of about 24 putative lipase/esterase genes, belonging to the so called “*lip*” family (*lipC* to *lipZ*), based on the presence of a consensus ‘GXSXG’ motif, a characteristic feature of the members of the α/β hydrolase-fold family^13^. However, only few of these proteins like LipC (Rv0220)^14^, LipF (Rv3487c)^15^, LipH (Rv1399c)^16^ and LipY (Rv3097c)^17^,^18^ are functionally characterized to some extent.

One of the PE family proteins, PE11 (LipX or Rv1169c) is found to be specifically present in pathogenic strains like *M. tuberculosis*, *M. bovis* and clinical strain *CDC1551* but is conspicuously absent in non-pathogenic *M. smegmatis* strain. PE11 is known to be upregulated in *M. tuberculosis* during acidic stress, starvation and adaptation to stationary phase as well as in hypoxic lipid-loaded macrophages^12^,^19^,^20^,^21^. Also its expression is found to be increased during palmitic acid induced stress conditions^17^. Presence of anti-PE11 antibody in TB patients^22^ and its up-regulation in human lung granulomas^23^ suggest that PE11 is possibly over-expressed during active tuberculosis infection. However, the exact functions of PE11 remains poorly understood. PE11 is predicted to be a putative lipase/esterase due to presence of ‘GXSXG’ motif, characteristic of the α/β hydrolase fold family^13^. However, whether PE11 is capable of modulating the cell wall architecture and lipid composition and whether these changes contribute to mycobacterial virulence are not well known. In this study, we demonstrate esterase activity of PE11 and its role in cell wall remodeling. Further, the data presented herein indicate that PE11 probably plays an important role in *M. tuberculosis* virulence and establishment of a successful infection.

## Results

### Expression of PE11 in *M. smegmatis* altered colony morphology and architecture of the bacilli

**Figure 1:**
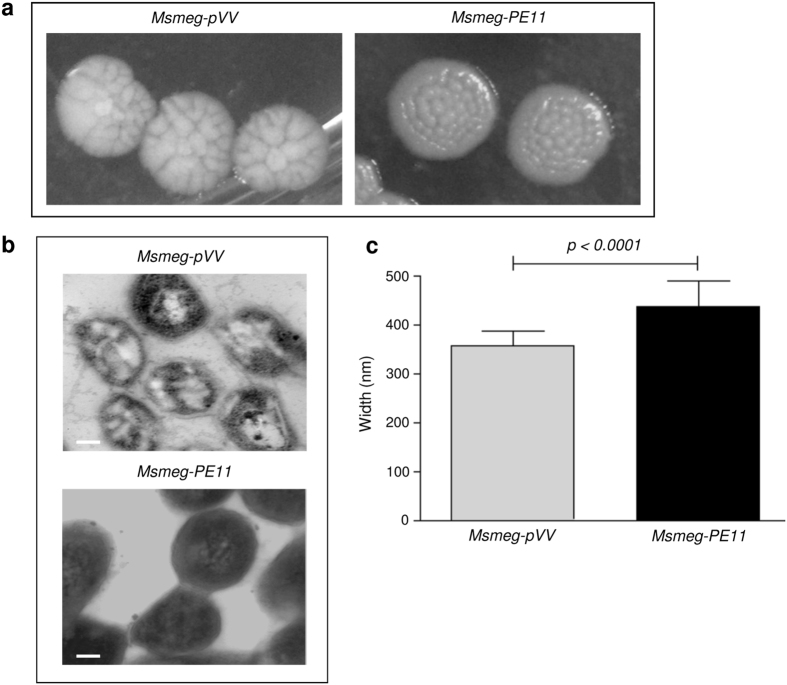
PE11 alters colony morphology, surface architecture and width of *M. smegmatis*. (**a**) *Msmeg-pVV* or *Msmeg-PE11* bacteria were plated on 7H10 agar plates supplemented with OADC and incubated for 5–6 days and the mycobacterial colonies were photographed. (**b**) For transmission electron microscopy (TEM) analysis, *Msmeg-pVV* or *Msmeg-PE11* strains were cultured on 7H10 agar for 5–6 days and surface architecture of these bacteria were analyzed. Scale bar, 100 nm. (**c**) In another experiment, *Msmeg-pVV* or *Msmeg-PE11* bacteria were harvested for scanning electron microscopy (SEM) analysis to measure the diameters of *Msmeg-pVV* or *Msmeg-PE11* and mean width of 100 bacilli each of *Msmeg-pVV* and *Msmeg-PE11* is graphically represented in nm.

To understand how PE11 influence mycobacterial pathophysiology, we expressed *pe11* gene in a non-pathogenic *M. smegmatis* strain mc^2^155 (*Msmeg-PE11*), which is widely used as a surrogate bacterium to characterize *M. tuberculosis* protein^24^. The immunoblot analysis indicates presence of PE11 in *Msmeg-PE11* but not in *M. smegmatis* harboring the backbone vector (*Msmeg-pVV*) (Supplementary Fig. 1). When the transformants were grown on Middlebrook 7H10 agar plates containing 0.5% glycerol, 10% OADC (oleic acid-albumin-dextrose [glucose]-catalase), and 0.05% Tween 80 and incubated for 5–6 days at 37 °C, we observed a distinct colony morphology in PE11 positive transformants. While the colonies of *Msmeg-pVV* were found to be usual irregular wrinkled acne-like structures, those of *Msmeg-PE11* were found to be rounded, shiny and smooth (Fig. 1a). Further, the control colonies were dry and fragile but the *Msmeg-PE11* colonies were wetter and stickier. Since, PE11 was predicted to be a putative lipase/esterase like protein^13^, our observations are suggestive of a role of PE11 in changing the cell wall components of *Msmeg-PE11*.

We next compared the surface architecture of *Msmeg-PE11* with *Msmeg-pVV* using transmission electron microscopy (TEM). In TEM analysis, we consistently observed poor contrast and hyper-staining of *Msmeg-PE11* cells compared to *Msmeg-pVV* (Fig. 1b), which is indicative of alteration in the cell wall composition of *M. smegmatis* expressing PE11. Examination of the bacteria under a scanning electron microscope indicated that expression of PE11 in *M. smegmatis* could alter the cellular dimension of the bacilli (Fig. 1c and Supplementary Fig. 2). The *Msmeg-PE11* cells were found to be significantly wider in diameter [437.2 ± 5.114 nm (mean ± SD)] as compared to *Msmeg-pVV* cells [356.1 ± 3.014 nm (mean ± SD)]. These results indicate that expression of PE11 alters the morphology and cellular dimensions of *M. smegmatis* bacilli.

### PE11 increases hydrophobicity of *M. smegmatis* cell envelope

**Figure 2:**
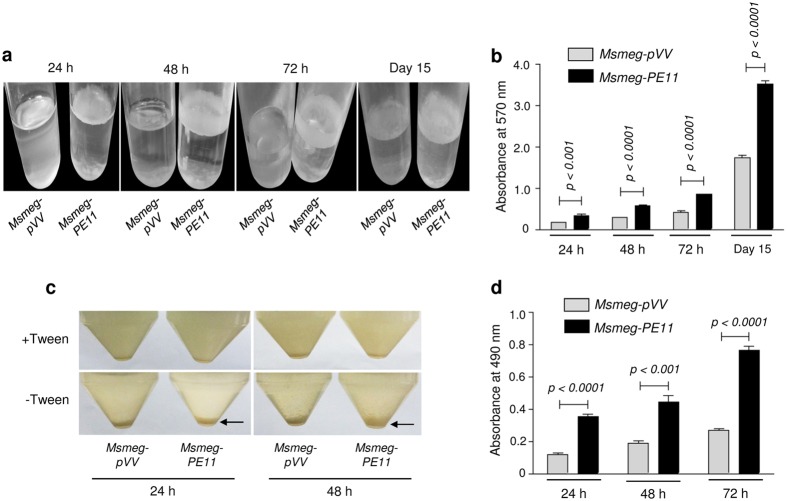
Cell surface of *Msmeg-PE11* is highly hydrophobic. (**a**) Pellicle formation of *Msmeg-pVV* or *Msmeg-PE11* was monitored by growing standing cultures of the strains without shaking in Middlebrook 7H9 medium in absence of Tween 80 at 37 °C for various time points. (**b**) Biofilm formation was quantified by crystal violet staining for which *Msmeg-pVV* or *Msmeg-PE11* cells were washed, stained with 1% crystal violet and ethanol extract was spectrophotometrically measured at 570 nm. (**c**) *Msmeg-pVV* or *Msmeg-PE11* were cultured in 7H9 medium with (upper panel) or without (lower panel) 0.05% Tween-80 at 37 °C with shaking for 24 h and 48 h. Cultures were then allowed to settle at room temperature for 30 min. (**d**) In another experiment, *Msmeg-pVV* or *Msmeg-PE11* were cultured in 7H9 broth with congo red (100 μg/ml) and 0.05% Tween 80 for 24 h, 48 h and 72 h at 37 °C. Cells were next washed and re-suspended in acetone. Congo red in the supernatant was spectrophotometrically measured at 490 nm. Data are representative of mean ± SD of three different experiments.

Several studies have proposed that changes in the cell wall lipid composition can significantly influence the surface hydrophobicity and various cell surface properties like biofilm/pellicle formation, congo red binding, cellular aggregation and sliding motility^25^,^26^. Since, earlier we observed conspicuously different colony morphology and increased electron-dense staining in TEM analysis, we next examined cell surface properties like cellular aggregation, pellicle/biofilm formation and congo red binding. When the pellicle formation at the air-liquid interface of the static 7H9 liquid cultures (without Tween 80) was examined as described elsewhere^27^, *Msmeg-pVV* was found to form thin visible surface pellicle, but cells from *Msmeg-PE11* were found to be heavily aggregated and were able to form thick and robust pellicles at the air-liquid interface under similar experimental conditions (Fig. 2a). The pellicles are believed to be biofilm-like structures that assemble at the air-liquid interface. The biofilm formation was quantified using crystal violet stain as described by others^27^. The results shown in Fig. 2b indicate that compared to the control *Msmeg-pVV*, biofilm formation was significantly increased in *Msmeg*-*PE11,* indicating a role of PE11 in biofilm formation and possible changes in the cell surface properties.

Detergent like Tween 80 (0.05% vol/vol) is added in liquid mycobacterial cultures to prevent clumping of bacterial cells. As expected, 24 h and 48 h old cultures of *Msmeg-pVV* and *Msmeg-PE11* remained completely dispersed in medium in the presence of Tween 80 (Fig. 2c, upper panel, all tubes). However, growth in liquid medium without Tween 80 resulted in cell aggregation that led to reduced turbidity in 24 h and 48 h old cultures of *Msmeg-pVV* (Fig. 2c, lower panel, left tube) as well as *Msmeg-PE11* (Fig. 2c, lower panel, right tubes) when allowed to settle at room temperature for 30 min. Interestingly, in *Msmeg-PE11* cultures, the cells were found to be more aggregative and precipitated quicker in standing cultures than *Msmeg-pVV* at both 24 h and 48 h time points (Fig. 2c, lower panel). More aggregation of the *Msmeg-PE11* cells in liquid cultures in the absence of Tween 80 is likely due to an increased surface hydrophobicity of these cells, indicating an alteration of the cell wall architecture in *Msmeg-PE11*.

To confirm the difference in hydrophobicity between *Msmeg-pVV* and *Msmeg-PE11*, congo red binding assay was carried out. Congo red, a planar and hydrophobic diazo-dye that binds to lipids, lipoproteins^28^, is widely used to characterize lipid content and modifications affecting the cell wall composition and hydrophobicity^25^,^28^. Cells from *Msmeg-PE11* were found to retain three fold more congo red dye as compared to *Msmeg-pVV* (Fig. 2d) further confirming that *Msmeg-PE11* cells had higher surface hydrophobicity.

### *Msmeg-PE11* cells are more resistant to cell wall stress

**Figure 3:**
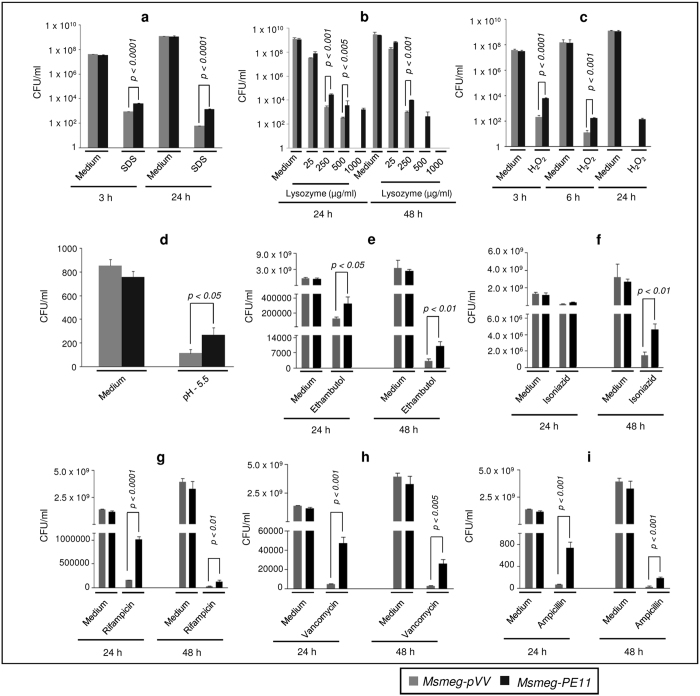
*Msmeg-PE11* are more resistant to SDS, lysozyme, hydrogen peroxide, low pH and antibiotics treatment when compared with *Msmeg-pVV*. The *Msmeg-pVV* or *Msmeg-PE11* were either treated with medium alone or subjected to (**a**) 0.1% SDS, (**b**) lysozyme, (**c**) 5 mM hydrogen peroxide, (**d**) low pH (5.5 for 24 h), (**e**) 20 μg/ml ethambutol, (**f**) 20 μg/ml isoniazid, (**g**) rifampicin (20 μg/ml), (**h**) vancomycin (5 μg/ml) and (**i**) ampicillin (750 μg/ml) treatment. Bacterial CFUs were counted on 7H10 agar plates. Data are representative of mean ± SD of three different experiments.

The mycobacterial cell wall is a rigid structure that enables the bacilli to survive under unfavorable intracellular environment. Conditions like low oxygen tension, chemical, oxidative, and acidic stress are encountered by *M. tuberculosis* during infection. Our earlier data indicate that PE11 is probably required to remodel cell wall architecture of mycobacteria. Since, PE11 is found to be present in the cell wall of virulent mycobacteria and is known to be upregulated during stress conditions^19^,^20^,^21^,^29^,^30^, it is possible that the protein plays an important role to protect the bacilli from various environmental stressors. Therefore, next we examined the ability of *Msmeg-PE11* to resist antibiotics and other stress conditions that mimic the intracellular environment. The *Msmeg-PE11* and the control *Msmeg-pVV* bacteria were either left untreated or treated for 3 h and 24 h with 0.1% SDS detergent that is known to disintegrate bacterial cell wall. Results shown in Fig. 3a suggest that *Msmeg-PE11* is more resistant to 0.1% SDS detergent treatment than the control *Msmeg-pVV* at both 3 h and 24 h time points. These findings suggest that PE11 confers resistance to detergent-mediated damage to mycobacterial cell envelope.

The antimicrobial agent lysozyme is secreted abundantly by macrophages, neutrophils, and lung epithelial cells. Lysozyme is known to damage mycobacterial cell wall by hydrolysing glycosidic bonds in peptidoglycan. When *Msmeg-pVV* and *Msmeg-PE11* were treated with various concentrations of lysozyme for 24 h and 48 h, it was observed that *Msmeg-PE11* was significantly more resistant to lysozyme treatment as compared to the control (Fig. 3b).

Next, we examined the ability of *Msmeg-PE11* bacteria to resist oxidative (hydrogen peroxide) and acidic stress (pH 5.5), as macrophages produce reactive oxygen species (ROS) and low pH environment as defense against *M. tuberculosis*. When *Msmeg-pVV* and *Msmeg-PE11* were treated with 5 mM hydrogen peroxide (H_2_O_2_) at various time points, it was found that *Msmeg-PE11* was more resistant to H_2_O_2_ than *Msmeg-pVV* (Fig. 3c). Also, after exposure to pH 5.5 for 24 h, survival of *Msmeg*-*PE11* was found to be 2.5 fold higher than the control *Msmeg-pVV* (Fig. 3d). We further evaluated the effect of PE11 on antibiotic sensitivity by comparing the survival of *Msmeg-pVV* and *Msmeg*-*PE11* strains after treatment with various antibiotics like ethambutol, isoniazid, rifampicin, vancomycin and ampicillin for 24 h and 48 h. The results shown in Fig. 3e–i indicate that *Msmeg-PE11* offered better resistance against all the drugs tested as compared to *M. smegmatis* harboring the vector control (*Msmeg-pVV*). We concluded that PE11-mediated changes in the cell wall architecture of *Msmeg-PE11* resulted in lesser penetration of antibiotics. Expectedly, *Msmeg-PE11* had better survival rates within primary macrophages (Supplementary Fig. 3). Taken together, these data indicate that PE11 may be important for maintaining a resilient cell wall structure which enables the bacteria to persist longer inside the macrophages.

### PE11 shows esterase activity

**Figure 4:**
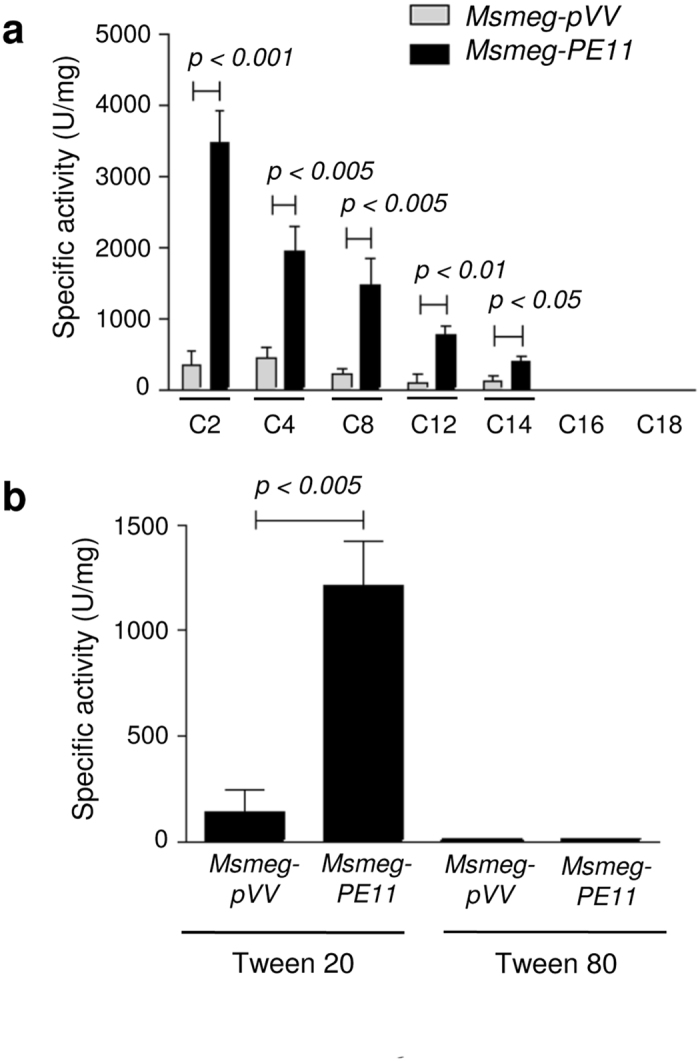
PE11 has esterase activity. (**a**) Lipid hydrolyzing activity of PE11 was determined by measuring the release of *para*-nitrophenol (pNP) upon incubation of 20 μg of cell lysate prepared from either *Msmeg-pVV* or *Msmeg-PE11* with *p*-nitrophenyl acetate (C2), *p*-nitrophenyl butyrate (C4), *p*-nitrophenyloctanoate (C8), *p*-nitrophenyldodecanoate (C12), *p*-nitrophenylmyristate (C14), *p*-nitrophenylpalmitate (C16) and *p*-nitrophenyl stearate (C18) at 37 °C and pH 7.0 for 10 min. The release of *para*-nitrophenol was measured spectrophotometrically at 405 nm. (**b**) Also, lipolytic activity of PE11 was monitored by cleavage of polyoxyethylene sorbitan monolaurate (Tween 20) and polyoxyethylene sorbitan monooleate (Tween 80) at pH 7.0. Data are representative of mean ± SD of three different experiments.

We next characterized the enzyme activity of PE11 using esters of *p*-nitrophenyl (pNP), *p*-nitrophenylacetate (C2), *p*-nitrophenylbutyrate (C4), *p*-nitrophenyloctanoate (C8), *p*-nitrophenyldodecanoate (C12), *p*-nitrophenylmyristate (C14) *p*-nitrophenylpalmitate (C16) and *p*-nitrophenylstearate (C18) as substrates. We found that lysates prepared from *Msmeg-PE11* had significantly higher hydrolyzing activities to a wide range of *para*-nitrophenyl (pNP) esters (C2–C14) with increased preferences to shorter carbon chain length derivatives at pH 7.0 and 37 °C (Fig. 4a). The pNP ester *para*-nitrophenylacetate containing the shortest carbon chain (C2) was found to be most efficiently hydrolyzed (Fig. 4a). Though significantly higher hydrolyzing activity was detected with intermediate carbon chain length pNP esters, no activity was found towards longer carbon chain pNP esters (Fig. 4a). These results suggest that PE11 protein is acting predominantly as an esterase rather than lipase. In contrast, the lysates prepared from *Msmeg-pVV* had very low hydrolyzing activity against C2–C14 pNP esters tested as compared to those prepared from *Msmeg-PE11* (Fig. 4a), indicating that the esterase activity of *Msmeg-PE11* lysate is attributable to PE11.

We further confirmed the esterase activity of PE11 by employing a turbidimetric esterase assay where the cleavage of polyoxyethylene sorbitans (Tweens) was measured^31^. This method involves hydrolysis of Tweens in the presence of CaCl_2_, and measuring the increased absorbance at 405 nm due to precipitation of the released fatty acids as calcium salts. Tween 80 is mostly hydrolyzed by lipase activity and rarely by esterase, as it contains esters of long chain oleic acid (C18). In contrast, Tween 20 is easily hydrolysable by an esterase as it contains esters of shorter chain lauric acid (C12)^31^. This assay is reported to be about 36-fold more sensitive than the titrimetric assay and at least 4-fold more sensitive than the colorimetric assay using pNP esters^32^. When *Msmeg-PE11* lysates were tested for hydrolysis of polyoxyethylene sorbitan monolaurate (Tween 20), about 9 fold higher lipolytic activity was recorded compared to the control *Msmeg-pVV* lysates (Fig. 4b). The level of lipolytic activity of *Msmeg-PE11* lysate on Tween 20 was 1215 ± 205.36 mU/mg at pH 7.0 whereas activity of the control lysate was limited to 138.33 ± 109.83 mU/mg. When polyoxyethylene sorbitan monooleate (Tween 80) was used as a substrate, negligible lipolytic activity was detected in both *Msmeg-PE11* and *Msmeg-pVV* lysates. The observed data were in concordant with the previous results obtained using pNP esters as substrate indicating that PE11 is predominantly an esterase.

### PE11 modulates the synthesis of glycolipids

**Figure 5:**
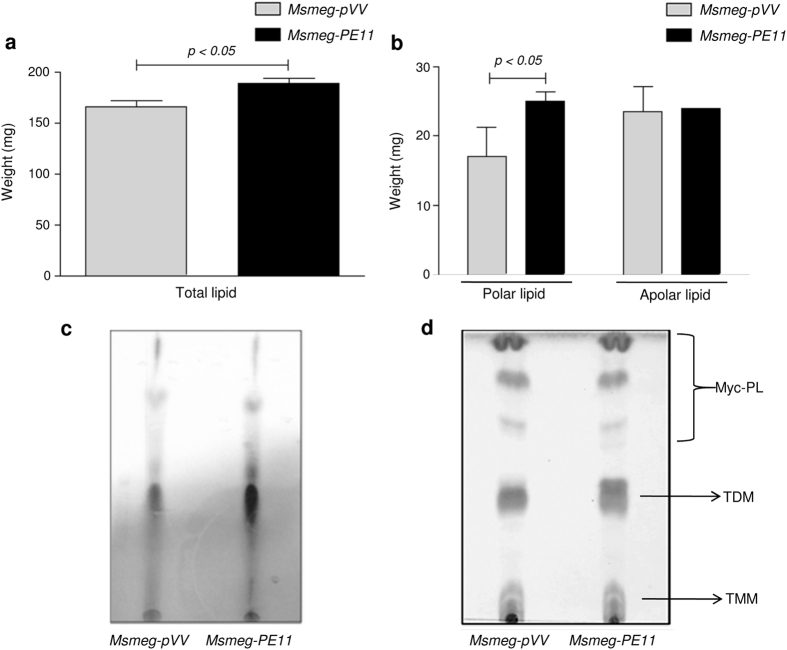
Modulation of total, polar and glycolipids in *Msmeg-PE11*. Quantification (% weight) of (**a**) total lipids, (**b**) polar and apolar lipids extracted from the *Msmeg-pVV* or *Msmeg-PE11* wet pellets (equal weights), with chloroform-methanol (2:1, vol/vol). (**c**) Total glycolipid analysis of *Msmeg-pVV* or *Msmeg-PE11* by analytical TLC. Methanol: Ammonium hydroxide (80:20, vol/vol) was used as mobile phase and the glycolipids were visualized by α-naphthol/sulfuric acid staining (**d**) Mycolic acid containing glycolipids were separated from the pool of total polar lipids using Chloroform:Methanol:Ammonium hydroxide (80:20:2, vol/vol/vol) as mobile phase. These glycolipids were identified by Iodine vaporization followed by charring with 5% phosphomolybdic acid.

The changes in colony morphology and various cell surface properties like cellular aggregation, pellicle/biofilm formation, congo red binding and increased resistance to various stressors, suggested that the presence of PE11 resulted in modulation of components of the cell wall lipids. In order to confirm this hypothesis, we analyzed the lipid content of *Msmeg-pVV* and *Msmeg*-*PE11* cells at the same stage of growth. *Msmeg*-*PE11* was found to have increased quantities of both total and polar lipids in the cell envelope (Fig. 5a,b), however, the apolar lipid content was not found to be significantly changed (Fig. 5b). Mycobacteria defective in glycopeptidolipid (GPL) biosynthesis is often found to possess altered colony morphologies^33^ and biofilm formation^26^,^34^. Therefore, we examined whether the glycolipid composition and production was changed in *M. smegmatis* due to PE11 expression. Thus, total cell envelope lipids were extracted and separated by TLC (Thin layer chromatography), and glycolipids were visualized by α-naphthol/sulfuric acid staining. Interestingly, we found that the expression of PE11 led to an increase in glycolipid concentration in the cell wall (Fig. 5c). We also analyzed the cell wall to compare mycolate containing glycolipids [trehalose monomycolate (TMM), trehalose dimycolate (TDM) and mycolylmannosylphosphorylheptaprenol (Myc-PL)]. TLC results indicate an increase of TDM in *Msmeg*-*PE11* cell wall (Fig. 5d). However, the relative amounts of TMM and Myc-PL were not significantly changed (Fig. 5d). These observations indicate that expression of PE11 led to an increased deposition of glycolipids in the cell wall of *M. smegmatis.*

### Quantitative modulation of fatty acid composition of *Msmeg-PE11*

Next, we analyzed the fatty acid composition of *Msmeg-pVV* and *Msmeg-PE11* strains using gas chromatography coupled with mass spectrometry (GC/MS). The total lipid fraction from each strain was transesterified and methylated, and the resulting fatty acid methyl esters (FAMEs) were analyzed by GC and GC/MS. GC chromatograms showed that the major FAMEs produced by *Msmeg-pVV* and the *Msmeg-PE11* were C16:0, C16:1, C18:0, C18:1 and C18:0 (10-methyl) FAMEs based on their retention times and molecular masses (Supplementary Fig. 4). Interestingly, we observed an increase in C18:0 (10-methyl) FAME representing branched fatty acids, there was approximately 36% more abundance of C18:0 (10-methyl) FAME in *Msmeg-PE11* from FAMEs isolated from total lipid and similar pattern was also observed in FAMEs isolated from polar lipid (Table 1). However, we noted 25% decrease in linear C18:0 FAME in *Msmeg-PE11* (Table 1). A cluster of fatty acids longer than the C20 were more in the *Msmeg-PE11* compared to *Msmeg-pVV*. These fatty acids were found to be between 18 and 24 min retention times (Supplementary Fig. 4 and Table 1). Further, these fatty acids mainly belong to a homologous series differing only in the acyl chain lengths. The identified FAMEs from total and polar lipid extracted from the *Msmeg-PE11* strain were further validated by GC/MS, using the NIST02.L database. In case of apolar lipids, analyses of fatty acid composition did not reveal much difference between *Msmeg*-*PE11* and *Msmeg-pVV* except there was approximately 27% more abundance of C24:0 FAME in *Msmeg-PE11.* Taken together, these data suggest that PE11 significantly modulated the fatty acid profiles extracted from the polar lipid of the cell wall.

### PE11 confers a growth advantage to *M. smegmatis* in a mouse model of infection

**Figure 6:**
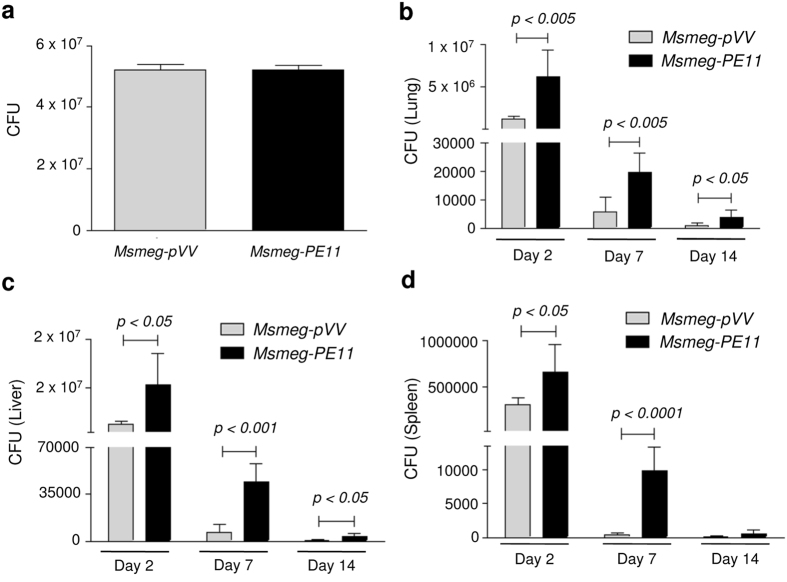
Better persistence of *M. smegmatis* expressing PE11 in mice. Balb/c mice (n = 6) were infected intravenously with approximately (**a**) equal number (5 × 10^7^) of *Msmeg-pVV* or *Msmeg-PE11*, and sacrificed after 2, 7 and 14 days post-infection for determination of CFU in (**b**) lung (**c**) liver and (**d**) spleen. Data represent mean ± SD of six mice per group for each time point.

We next assessed the virulence property of PE11 *in vivo* using an animal infection model. The Balb/c mice were infected with 5 × 10^7^ CFU of either *Msmeg-pVV* or *Msmeg*-*PE11* via lateral tail vein route and the bacterial burden was estimated in lung, liver and spleen of the infected animals at day 2, 7 and 14 post-infection. Both *Msmeg-pVV* and *Msmeg-PE11* were dispersed into single cell suspensions and plated to confirm administration of equal number of bacteria in mice (Fig. 6a). The CFU counts indicated that *M. smegmatis* expressing PE11 persisted significantly for longer time period than *M. smegmatis* harboring the pVV vector in all the organs at almost all the time points investigated. In lungs of *Msmeg-pVV*-infected mice, the mean CFU counts were significantly lower at day 2 post-infection as compared to those of infected with *Msmeg-PE11* strain and this trend also continued to later time points (day 7 and 14) (Fig. 6b). Similar observations were also found in liver (Fig. 6c) as well as in spleen (Fig. 6d). These results indicate that expression of PE11 protein in non-pathogenic mycobacteria results in longer survival of the bacilli in host cells signifying that PE11 probably plays a role in mycobacterial virulence.

**Figure 7:**
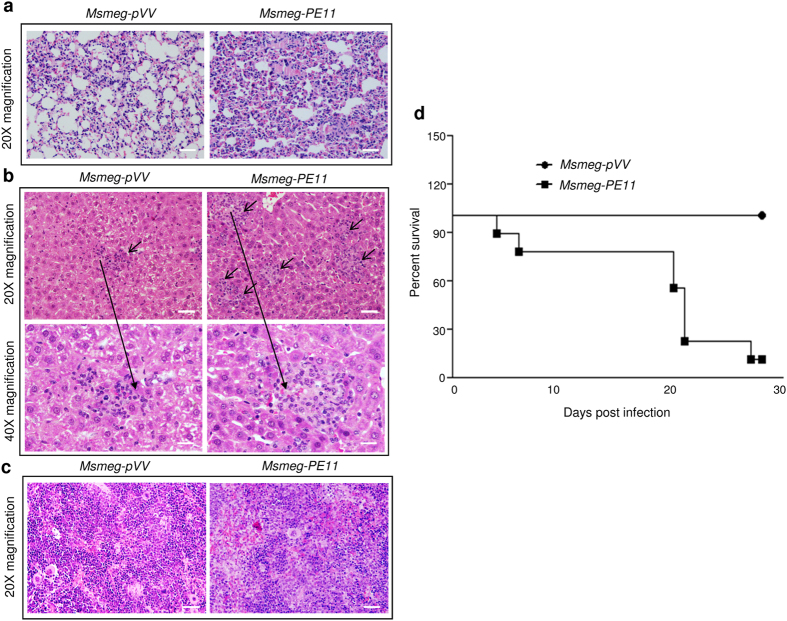
Balb/c mice infected with *Msmeg-PE11* showed more organ pathology and decreased survival rate as compared to mice infected with *Msmeg-pVV*. The lung (**a**), liver (**b**) and spleen (**c**) sections of Balb/c mice infected with 5 × 10^7^ of *Msmeg-pVV* or *Msmeg-PE11* were stained with hematoxylin and eosin (H&E) stain at day 7 post-infection. The microgranuloma like structure in liver sections is marked with arrows and one of these is shown at 40X magnification. Photographs of representative sections from 2 mice were shown. Scale bar, 200 μm for lung, liver and spleen. (**d**) Survival of Balb/c mice (n = 10) following intravenous infection with 5 × 10^7^ of *Msmeg-pVV* or *Msmeg-PE11* was recorded for 30 days post-infection.

Mycobacterial burden in mice organs is generally well correlated with tissue pathology^35^,^36^. Therefore, we evaluated the extent of tissue damage in lung, liver and spleen in mice infected with *Msmeg-PE11* or *Msmeg-pVV* strains by histopathological examination of hematoxylin and eosin (H&E) stained sections. It was found that *Msmeg-PE11*-infected mice had markedly pronounced tissue damage than that observed in *Msmeg-pVV*-infected mice (Fig. 7). Infiltration and lesions were more severe in the lungs of mice infected with *Msmeg-PE11* by day 7 post-infection (Fig. 7a). Furthermore, extensive tissue necrosis, increased lymphocytic and phagocytic cellular infiltration and disappearance of alveolar spaces were recorded in the lungs of *Msmeg-PE11-*infected mice compared to lungs of *Msmeg-pVV*-infected mice. A similar pathology was also observed in the liver of *Msmeg-PE11-*infected mice. The lesions were found to be more severe with increased microgranuloma and more lymphohistiocytic in mice infected with *Msmeg-PE11* than that of mice infected with *Msmeg-pVV* (Fig. 7b). Liver sections from *Msmeg-pVV-* infected mice showed mild lymphocytic infiltration and microgranuloma at day 7 post-infection and this was correlated well with lower CFU counts in the liver. Additionally, single cell necrosis began to appear in the liver of *Msmeg-PE11-*infected mice by day 7 post-infection (Fig. 7b). The histopathological analyses of spleen indicated presence of more white pulp in *Msmeg-PE11*-infected mice sacrificed at day 7 (Fig. 7c) and day 14 (Supplementary Fig. 5) post-infection. Also, there was an increase in the size and weight of spleen in *Msmeg-PE11*-infected mice as compared to *Msmeg-pVV*-infected mice sacrificed at day 14 (Supplementary Fig. 6a,b). To inquest the effect of *PE11* on mortality, survival of Balb/c mice infected with *Msmeg-pVV* and *Msmeg-PE11* was monitored. No deaths were registered in the group of mice infected with *Msmeg-pVV* strain during the entire study period of 30 days. However, in the group of mice infected with *Msmeg-PE11*, survival rate was found to be dropped to 20% by 28 days post-infection (Fig. 7d). Also, mice infected with *Msmeg-PE11* had significant decrease in the total body weight at day 9 and day 18 post-infection as compared to mice infected with *Msmeg-pVV* (Supplementary Fig. 7). By day 27, mice infected with *Msmeg-pVV* returned to the baseline value whereas most of the *Msmeg-PE11*-infected mice were found to be dead.

### PE11 induces T-helper 2 (Th2) cytokines in addition to tumor necrosis factor-α (TNF-α) in lung and liver of infected mice

**Figure 8:**
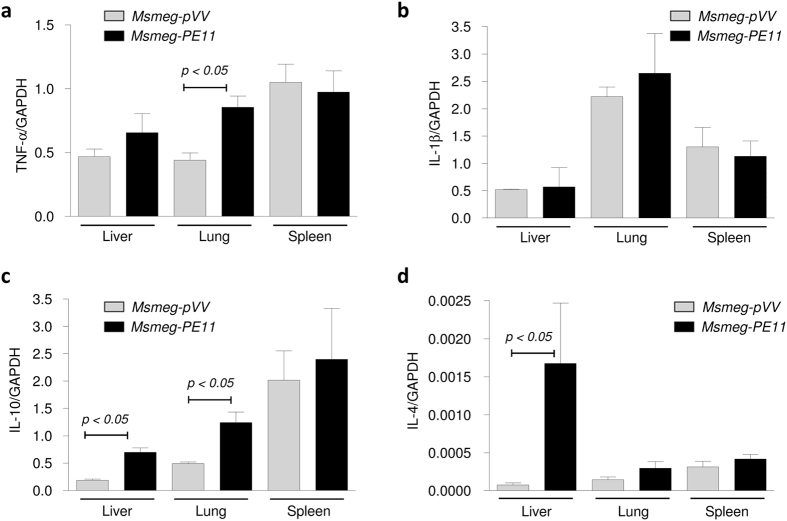
Cytokine profile of Balb/c mice infected with *Msmeg-PE11* and *Msmeg-pVV* at day 7 post-infection. Balb/c mice were infected intravenously with 5 × 10^7^ *Msmeg-PE11* and *Msmeg-pVV* and at day 7 post-infection, the mice were sacrificed and total RNA was isolated from liver, lung and spleen. The levels of TNF-α (**a**), IL-1β (**b**), IL-10 (**c**) and IL-4 (**d**) in different tissues were measured by reverse-transcription qPCR and expression levels were normalized to corresponding GAPDH levels. The data are representative of 3 mice in each group.

The levels of proinflammatory cytokines like TNF-α and interleukin (IL)-1β and Th2 cytokines like IL-10 and IL-4 were measured by reverse transcription qPCR (RT-qPCR) in the liver, lung and spleen of the infected mice at day 7 post-infection. It was found that the levels of TNF-α were considerably higher in liver and lungs (Fig. 8a) in the mice infected with *Msmeg-PE11* as compared to those of mice infected with *Msmeg-pVV*, however, IL-1β levels did not differ significantly both at transcript and protein levels (Fig. 8B and Supplementary Fig. 8). On the other hand, the levels of IL-10 (Fig. 8c) was found to be significantly higher in both lungs and liver of the mice infected with *Msmeg-PE11*as compared to those infected with control *Msmeg-pVV*. Similarly, IL-4 cytokine levels were also found to be considerably higher in liver and lungs of mice infected with *Msmeg-PE11* (Fig. 8d). Interestingly, none of the cytokines were found to differ significantly in the spleen of *Msmeg-PE11*-infected mice at day 7 post-infection.

## Discussion

The unique cell envelope of *M. tuberculosis* has been implicated in the pathogenesis and resistance to anti-mycobacterial drugs. In the present study, we attempted to understand the possible role played by a Lip family protein of *M. tuberculosis,* PE11 which is also a member of the unique PE/PPE family proteins, in mycobacterial pathophysiology and virulence. PE11 is known to be induced under several stressful conditions those are frequently encountered by the invading *M. tuberculosis* inside the hostile cellular environment. Microarray analyses of the transcriptome responses under acidic conditions found in phagosomes revealed that a number of genes including those involved in fatty acid metabolism including *pe11* were upregulated, possibly required for long term survival under such conditions^19^. *pe11* is also found to be upregulated during nutrient starvation^30^ as well as in hypoxic conditions^37^ and in response to H_2_O_2_ treatment in *M. tuberculosis*^38^. In addition, *pe11* is also found to be upregulated in response to stressors like SDS and diamide^39^,^40^. Further, data on the transcriptional responses of *M. tuberculosis* to bovine lung surfactant has shown that *pe11* is significantly induced^41^. Also, *pe11* is shown to be induced during non-replicating persistent conditions^21^. PE11 transcripts are known to be upregulated during macrophage infection and in granulomas of lungs of human pulmonary TB patients^20^,^23^,^42^. These data indicate that PE11 in conjunction with other lipases probably plays a role in release of fatty acids from triglycerides to be used as an energy source and may help the bacilli to adapt to the intracellular environment during infection and dormancy.

Bioinformatics analyses suggested that PE11 is a putative lipase/esterase^13^. From the published reports by at least two research groups, it appears that *Rv1169c* (*pe11*) is an essential gene for *in vitro* growth of *M. tuberculosis*^43^,^44^. Therefore, we used *M. smegmatis*, a widely used surrogate bacterium to investigate the pathophysiological role of PE11 *in vitro* and *in vivo*. We found that PE11 could significantly modify cell surface properties when expressed in *M. smegmatis*, and these modifications might be explained by its ability to alter the cell wall lipid composition. Sequence analysis of PE11 predicted presence of the ‘α/β hydrolase fold’ characteristic of lipase/esterase family of proteins. Biochemical assays of PE11 using a wide range of *p*-nitrophenyl esters with varying carbon chain lengths from C2 to C18 clearly showed that PE11 possesses esterase activity that could preferentially hydrolyzes shorter (C2–C4) to intermediate (C8–C14) carbon chain esters but failed to hydrolyze longer chain *p*-nitrophenyl esters (C16–C18) indicating that PE11 is predominantly an esterase rather than a lipase. The *p*-nitrophenyl acetate (C2) was found to be most efficiently hydrolyzed by PE11. The turbidimetric esterase assay using Tween 20 and Tween 80 as its substrates further confirmed the esterase activity of PE11. Interestingly, another protein of *M. tuberculosis*, Rv0045c was also reported to be an esterase^45^ that also hydrolyzes short chain *p*-nitrophenyl esters (C2–C8), but its preferred substrate was *p*-nitrophenyl caproate (C6). Similarly, another PE family protein, PE16 (Rv1430) could hydrolyze a wide range of *p-*nitrophenyl esters (C4–C12), of which pNPC6 was found to be most effectively hydrolyzed^46^. On the other hand, the lipase enzyme LipY is shown to hydrolyze mainly the triacylglycerol (TAG)^17^. It is believed that the host TAG serves as the power house for *M. tuberculosis* and gets catabolized by the *M. tuberculosis* lipolytic enzymes sequentially into DAG (diacylglycerol), then to MAG (monoacylglycerol) which is finally hydrolyzed to release free fatty acids to be used as the carbon source for energy as well as synthesis of cell wall components^13^. Thus, esterases play an important role in the *M. tuberculosis* pathophysiology as the free fatty acids act as building blocks for maintenance of *M. tuberculosis* cell wall in hostile environment and also act as energy source for persistence and reactivation. Since, PE11 specifically hydrolyzes shorter carbon chain pNP ester, we presumed that it probably acts on the final hydrolyzed product of TAG, i.e., MAG for release of free fatty acids. Our observations of increased total lipid content in *Msmeg-PE11* cell wall as compared to the control *Msmeg-pVV* cell wall supports the hypothesis that PE11 is actually involved in mycobacterial lipid metabolism. Our data indicate that PE11 plays a structural role in modifying envelope composition through its esterase activity. Pertinent to this, PE11 appears to be capable of localizing on to the mycobacterial cell wall^29^. Therefore, it is tempting to speculate that the tubercle bacilli may employ PE11 for foraging and hydrolyzing shorter carbon chain lipids into fatty acids sourced from the local environment to be used for its energy requirement and structural function. Further, expression of PE11 during nutrient starvation^30^ suggests that PE11 might be required to maintain viability under nutritional stress.

Interestingly, Rv3036c of *M. tuberculosis* was also characterized as a novel cell wall-anchored esterase that hydrolyzes soluble *p*-nitrophenyl acetate^47^. However, its role in mycobacterial virulence and pathogenesis is not yet known. In this study, using a surrogate bacterium, we provide evidences that expression of PE11 can confer survival advantage to the bacilli inside the macrophages and further *in vivo* studies in a mice infection model supports a role for this protein in the mycobacterial pathogenesis and virulence. A putative carboxyl esterase, Rv2224c that preferentially hydrolyses ester bonds of substrates with intermediate carbon chain length is also found to be membrane associated and mutants had reduced pathology in mice lung indicating its role in the virulence of *M. tuberculosis*^48^. Also, LipY is demonstrated to play roles in *M. tuberculosis* virulence^18^, though the mechanisms are not clear. All these reports suggest an important role of the esterase/lipase in mycobacterial virulence.

Colony morphology of the mycobacterium species is known to be a complex phenotype associated with mycobacterial virulence. Colony morphology is also correlated to antibiotic susceptibility^49^, cytokine induction and MAP kinase signaling^50^,^51^. Earlier studies have indicated that changes in colony morphology are indicative of metabolic changes and virulence properties of *M. avium*^52^. Expression of *pe11* gene in *M. smegmatis* led to a shift from a usual rough to unusual smooth morphology of the bacilli is suggestive of a structural role of this protein. *Msmeg-PE11* cells were found to be wider in diameter as compared to *Msmeg-pVV* cells. We further observed an electron-dense staining of *M. smegmatis* expressing PE11 in TEM analysis. This implies that the changes in the composition and content of the surface exposed lipids of *M. smegmatis* due to expression of PE11 reacted more vigorously with uranyl acetate and lead citrate to give the more electron-dense appearance of the outermost cell envelope layer.

Changes in the cell wall lipid composition can also reflect marked variations in mycobacterial cell surface properties like pellicle/biofilm formation, congo red binding, cellular aggregation and sliding motility^25^,^26^,^27^. Mycobacteria grown in the absence of detergent tend to form surface pellicle, a biofilm like structure at the liquid-air interface when grown in synthetic media^53^ which can confer drug resistance and often harbor drug tolerant cells^54^. Pellicle formation may be regarded as an adaptive diversification of the organism in response to certain stimuli into a structured environment like biofilms which are considered to be surface-associated bacterial communities comprising micro colonies surrounded by extracellular matrix. We found that *M. smegmatis* expressing PE11 was able to form profuse pellicle as compared to the control cells (*Msmeg-pVV)*. Similarly, PE11 was found to increase the cell surface hydrophobicity causing an increased tendency of *Msmeg-PE11* to form cellular aggregates possibly due to an increase in the glycopeptidolipid content in the cell wall. We further found that *Msmeg-PE11* is more resistant to antibiotics as well as various stressors like SDS, lysozyme, H_2_O_2_ and low pH (5.5) those mimicking the hostile macrophages environments encountered by the bacilli^55^. Thus, our data suggest that PE11 is actively involved in the cell wall remodeling that may confer increased drug resistance and survival advantages to the mycobacteria inside macrophages. Recently, Deng *et al.*, (2015) also reported that PE11 (Rv1169c) can alter the composition of mycobacterial cell wall when expressed in *M. smegmatis*^56^. Interestingly, we found a different fatty acid profile from that reported by Deng *et al.*, (2015). Also, we observed an increased resistance of the bacteria expressing PE11 to various environmental stressors and antibiotics in contrast to the report by Deng *et al.*, (2015). This may be due to use of different promoters in different expression vectors as well as different positions of the Histidine tag used while expressing PE11 in *M. smegmatis*.

Previous studies have shown that changes in cell hydrophobicity and cell wall integrity are related to alteration in the lipid composition of the mycobacterium cell envelope^27^,^52^. In this context, the glycopeptidolipids (GPLs), which are species-specific glycolipids, are characterized by a high variability in glycosylation patterns. GPLs are present on the cell wall surface of pathogenic and non-pathogenic mycobacteria and are implicated to play critical roles in the colony morphology. Glycosylation of the lipopeptide core of GPLs is found to be a prerequisite for colony smoothness of the members of the *M. avium* complex^33^. In addition, the *mps* mutants of *M. smegmatis* which are devoid of GPLs also exhibited a rough colony morphotype^33^. Interestingly, we observed enhanced synthesis of GPLs in *Msmeg-PE11*, which at least partly explains the observed smooth colony morphology and altered surface properties in these cells. Interestingly, when we quantified the cell wall fatty acids as methyl esters (FAMEs) using a high throughput gas chromatography coupled with mass spectrometry (GC/MS), we found almost similar fatty acid composition in both the strains, however, expression of PE11 caused a noticeable decrease in the amount of linear C18:0 polar fatty acids (Table 1), along with an increase in the branched chain polar fatty acid content (C18:10-methyl) which may increase the membrane fluidity and the ability of *Msmeg-PE11* to tolerate environmental stress. Interestingly, branched chain fatty acids have been implicated in the development of pH-stress tolerance in *Listeria* monocytogenes^57^. From our lipid analysis data (Fig. 5), it is apparent that PE11 also significantly increased total polar lipid content in *M. smegmatis* without significantly affecting the total apolar lipid content. This may well be correlated with the observed persistence and virulence of *Msmeg-PE11* in mice infection models. The polar lipid fractions of pathogenic *M. bovis* were found to induce higher IL-10 production and antigen presenting cells had significant decrease in the levels of costimulatory molecules associated with antigen presentation including MHC-II and CD86^58^. In fact, we were able to detect higher levels of IL-10 and IL-4 cytokines in the liver and lung of *Msmeg-PE11* infected mice as compared to those infected with control *Msmeg-pVV* (Fig. 8c,d). On the other hand, *Msmeg-PE11* infected mice had higher induction of pro-inflammatory cytokines like TNF-α in the lung and liver (Fig. 8a) though the levels of IL-1β did not significantly differ (Fig. 8b and Supplementary Fig. 8). The high expression of IL-4 has been implicated as a virulence factor, both for its anti-inflammatory ability and also for its apparent capacity to promote tissue damage in association with TNF-α^59^. These observations are well correlated with the exacerbated pathology found in liver and lung and consequently mortality in *Msmeg-PE11*-infected mice similar to those observed in a Balb/c mouse model of pulmonary tuberculosis infection where appearance of IL-4 in the lung lesions coincides temporally and spatially with the appearance of areas of pneumonia and necrosis, leading to rapid clinical deterioration and death^60^.

In summary, we found that expression of PE11 in a non-pathogenic surrogate *M. smegmatis* could confer its properties akin to typical virulent mycobacteria including increased cell wall integrity, resistance to environmental stress, improved survival inside macrophages and mice. PE11 actively participates in remodeling the cell wall and may also provide energy by hydrolyzing acyl esters through its esterase activity for intracellular persistence of the pathogen. The possible localization of the *M. tuberculosis* PE11 at the cell surface^29^ and its involvement in the mycobacterial cell wall alteration prompted us to speculate that this protein may also participate in the active modulation of the immune responses. Accordingly, we found that PE11 can induce a pronounced Th2 environment by inducing cytokines like IL-10 and IL-4 in the lung and liver of mice infected with *M. smegmatis* expressing PE11, in addition to TNF-α which correlated well with the observed pathology in these tissues and also explains their higher CFU as compared to mice infected with control strain harboring the vector alone. Our findings suggest PE11 is a putative virulence factor for *M. tuberculosis* and may represent a promising target for future development of anti-mycobacterial therapeutics.

## Methods

### *M. smegmatis* culture and transformation

*M. smegmatis* mc^2^155 bacteria were grown in Middlebrook 7H9 medium (BD Difco, USA) supplemented with 10% OADC (HiMedia, India), 0.5% glycerol, and 0.05% Tween 80 (Fisher Scientific, USA). For preparing competent cells, the culture was allowed to grow until mid-log phase. The culture was centrifuged at 3000 rpm for 10 min, washed four times with 10% glycerol, and re-suspended in 1/100th of the culture volume in sterile deionized water with 10% glycerol and 100 μl aliquots of the cells were snap-frozen and stored at −80 °C until further use. Prior to transformation, the cells were thawed on ice, and 1 μg of DNA was added. The cells were incubated on ice for 10 min and transferred to a pre-chilled 1 mm gap width cuvette. One ml of the culture medium was added immediately, and the cells were incubated at 37 °C for 4 h. The transformants were selected on 7H10 (BD Difco, USA) agar plates containing 50 μg/ml kanamycin (Amresco, USA) and 50 μg/ml hygromycin B (Invitrogen, USA).

### Generation of recombinant *M. smegmatis* expressing PE11

For generation of recombinant *M. smegmatis* expressing PE11, the *pe11* gene was PCR amplified from *M. tuberculosis* H37Rv genomic DNA using specific primers (forward primer, F-5′-AATCGTGCATATGTCTTTTGTCACCACACGGCCCGATTC-3′ and reverse primer, R-5′CGGGATCCGGTGGAGGTGCCCGCGCGG-3′) encompassing the *pe11* ORF. The PCR amplicon was cloned into the NdeI and BamHI sites of pVV16 expression vector harbouring hsp60 promoter system (*pVV-pe11*) with a C-terminal His-tag. All the clones were confirmed by restriction digestion and the sequence of the inserts was validated by automated DNA sequencing. *M. smegmatis* underwent electroporation with *pVV-pe11* to generate recombinant *M. smegmatis* (*Msmeg-PE11*) and transformants were selected on 7H10 agar plate containing 10% OADC, 25 μg/ml kanamycin and 50 μg/ml hygromycin B. Expression of PE11 was confirmed by immunoblotting using anti-Histidine-tag antibody (Sigma-Aldrich, USA). *M. smegmatis* transformed with pVV16 vector (*Msmeg-pVV*) was used as control group.

### Preparation of *M. smegmatis* cell lysate

The *Msmeg-pVV and Msmeg-PE11* cultures were grown in Middlebrook 7H9 medium supplemented with 10% OADC, 0.5% of glycerol and 0.05% Tween 80 till the absorbance at 600 nm reached 0.6. Bacterial cell pellets were harvested and washed thrice with phosphate buffer saline with Tween 80 (PBS-T). The resulting pellets were then re-suspended in lysis buffer (50 mM Tris-Cl of pH 8.0, 300 mM NaCl and protease inhibitor) and vortexed. Bacterial cells were lysed using an ultrasonic sonicator and the lysed cells were centrifuged at 14000 rpm to collect the supernatant. Protein concentration was estimated using bicinchoninic acid method (Thermo scientific, USA).

### Reverse Transcription qPCR for cytokine

Total RNA was isolated from the tissues using TRI Reagent (Sigma-Aldrich, USA) as per manufacturer’s instructions. Briefly, the tissues were homogenized for 30 second in 0.5 ml TRI Reagent using a Mini BeadBeater (BioSpec Products, Inc., USA) and the aqueous phase was separated by addition of 0.1 ml chloroform. The total RNA in the aqueous phase was precipitated by addition of equal volume of isopropanol and the RNA pellet was washed with 75% chilled ethanol. After air drying the pellet was dissolved in RNAse-free water and treated with amplification grade DNAse I (Sigma-Aldrich, USA) to remove any genomic DNA contamination.

RNA concentration and purity was assessed using a Nanodrop ND-1000 spectrophotometer (Nanodrop Technologies,USA). RNA integrity was assessed using the RNA 6000 Nano LabChip Series II Assay with the 2100 Bioanalyzer System (Agilent Technologies, USA). About 2 μg total RNA with RIN (RNA integrity number) value of >7.5 were used for generation of first strand cDNA using First Strand cDNA synthesis kit (Thermo Scientific, USA) and using OligodT_18_ primers as per manufacturer’s instructions. The cDNA reactions were diluted to 50 μl in nuclease-free water, aliquoted and stored at −20 °C till further use.

Realtime qPCR reactions were carried out in duplicate for each sample using SYBR Premix Ex Taq (Tli RNaseH Plus, DSS Takara Bio India Pvt. Ltd, India) in a 20 μl reaction volume containing each primers at 500 nM final concentration. Reactions were temperature cycled, and SYBR green levels were measured using a CFX-96 system (Bio-Rad Laboratories, USA). The initial target concentration for each gene except IL-4 was calculated by relative standard curve method using a pool of experimental sample cDNA as calibrator. For IL-4, the calibrator for the relative standard curve was prepared by using a mixture of purified IL-4 and GAPDH PCR products in an equimolar ratio due to its low copy number and low levels of expression in most of the samples. The level of expression for each gene in each sample was normalized to corresponding GAPDH levels. All the primers were synthesized from Eurofins Genomics India Pvt Ltd (Bangalore, India). The sequences of the primers used for RT-qPCR were given in Supplementary Fig. 9.

### Colony morphology

For colony morphology, *Msmeg-pVV* and *Msmeg-PE11* strains were plated onto Middlebrook 7H10 (BD, Difco, USA) plates with 0.5% glycerol, and 10% OADC and incubated for 5–6 days at 37 °C. A Canon PowerShot G11 Digital camera (Japan) was used to take photomicrographs of colonies.

### Enzyme activity

The enzyme activity of PE11 was measured, as described elsewhere^45^, using different esters of *p*-nitrophenol (pNP) (Sigma-Aldrich, USA) such as acetate (pNPC2), butyrate (pNPC4), octanoate (pNPC8), laurate (pNPC12), myristate (pNPC14), palimitate (pNPC16) and stearate (pNPC18). Enzymatic reactions were performed at 37 °C for 10 min in a 100 mM Tris-HCl buffer (pH 7.0) containing mycobacterial cell lysate (20 μg) and 2 mM of pNP substrate, solubilized in acetonitrile (ACN). Final volume was fixed to 200 μl in each microtiter well. The enzymatic hydrolysis was quantified by measuring the absorbance of the hydrolyzed products of all the substrates (*p*-nitrophenol) at 405 nm using a 96-well plate spectrophotometer (Ultra Microplate Reader EL808, Bio-Tek, USA) using a standard pNP calibration curve. Activity was expressed in International units (U, corresponding to 1 μmol of pNP released per minute). Specific activities were expressed as U/mg of cell lysate. Lipolytic activity as determined by Tween cleavage was performed as previously reported^31^. Briefly, a reaction mixture was prepared in Tris (50 mM) buffer containing 33 mM CaCl_2_ and 0.33% Tween 20 (Ameresco, USA) or 0.33% Tween 80 (pH 7.0) and 40 μg cell lysate and incubated at 37 °C. Reaction mixture (200 μl) was transferred to a 96-well microwell plate, and turbidity was assessed by measuring the absorbance at 405 nm. Activity in this assay is reported as U/mg of lysate, where 1U is defined as the amount of enzyme required to induce a change in absorbance (405 nm) of 0.01 under assay conditions.

### Transmission electron microscopy (TEM)

For, TEM, cells were washed, fixed with 3% glutaraldehyde in 0.1 M phosphate buffer (pH 7.2) for 24 h at 4 °C and then fixed with 1% osmium tetroxide, dehydrated in a graded series of alcohol, and embedded in araldite 6005 resin. Ultra-thin sections (50–70 nm) were made with a glass knife on ultra-microtome (Leica Ultra cut UCT-GA-D/E-1/00), mounted on a copper grid, and stained with saturated uranyl acetate and counter stained with Reynold’s lead citrate. Specimens were examined using a Hitachi, H-7500 transmission electron microscope.

### Scanning electron microscopy (SEM)

For scanning electron microscopy, the *Msmeg-PE11* or Msmeg-*pVV* bacilli were harvested by low-speed centrifugation when the absorbance was about 0.6 at 600 nm. The bacterial pellets were washed in sterile PBS, and fixed for 24 h at 4 °C in a modified Karnovsky’s fixative in which cacodylate buffer was replaced with 0.2 M phoshphate buffer pH 7.4. Cells were then dehydrated through series of graded ethanol (30%, 50%, 70%, 80%, 90% and 100%) and dried by vacuum desiccation. The samples were then attached to stubs, sputter coated with gold (Model E-1010, Hitachi, Japan) and were examined using a Hitachi S3400N Scanning Electron Microscope at 15.0 kV. The diameter of the bacteria was measured by using ImageJ, an image analysis software available in the public domain.

### Mycobacterial cell surface properties

Mycobacterial cell surface properties were determined as described elsewhere^25^. For studying cellular aggregation, the bacilli were grown to an absorbance of 0.6 at 600 nm in 7H9 medium with or without 0.05% Tween 80 in a 37 °C shaker incubator. The cultures were kept at room temperature for 30 min to allow the cell aggregates to settle down, which were photographed with Canon PowerShot G11 Digital camera (Japan).

An assay for pellicle and biofilm formation was carried out as described earlier^26^,^27^. Briefly, pellicle formation was monitored by growing the cultures of *M. smegmatis* without shaking at 37 °C for 24 h, 48 h, 72 h and day 15 in Middlebrook 7H9 medium devoid of Tween 80 and the cultures were then photographed. To measure *M. smegmatis* biofilm formation, 200 μl of medium was added to 96-well polystyrene Costar plate (Corning, NY, USA) and inoculated with mycobacterial cells to an absorbance of 0.02 at 600 nm. The plates were then incubated at 37 °C for 24 h, 48 h, 72 h and day 15 for formation of biofilm. Next, the medium was discarded and the biofilm was washed with deionized water and stained with 1% crystal violet (Fisher Scientific, USA) for 30 min. Quantitation of biofilm formation was performed by extracting the biofilm associated crystal violet with 100% ethanol for 1 h and measuring the optical density at 570 nm. Congo red accumulation was determined by cultivating *Msmeg-pVV* or *Msmeg-PE11* for 24 h, 48 h and 72 h at 37 °C with shaking (200 rpm) in 7H9 broth in presence of 100 μg/ml congo red (Sigma-Aldrich, USA) and 0.05% Tween 80. Cells were collected by centrifugation (3000 g, 30 min) and washed extensively with distilled water until the supernatant was colorless. Cells were re-suspended in 1 ml acetone, vortexed and gently shaken for 2 h at room temperature, cells were then removed by high speed microcentrifugation and congo red in the supernatants was quantified spectrophotometrically by measuring absorbance at 488 nm (Ultra Microplate Reader EL808, Bio-Tek, USA).

### Measurement of sensitivity to SDS, lysozyme, hydrogen peroxide, low pH and antibiotics

Bacterial cultures were centrifuged and washed with 7H9 medium containing 0.05% Tween 80. Mycobacterial cell concentration was adjusted to an absorbance of 0.02 at 600 nm. To measure sensitivity towards low pH (pH 5.5), the bacteria were inoculated in 7H9 medium, pH 5.5 for 24 h at 37 °C. Sensitivity of bacterial culture towards 0.1% SDS (Sigma-Aldrich, USA) for 3 h and 24 h, and lysozyme (Sigma-Aldrich, USA) (25 μg/ml, 250 μg/ml, 500 μg/ml and 1000 μg/ml) for 24 h and 48 h, was measured at 37 °C. Further, to measure sensitivity to hydrogen peroxide, the bacteria were incubated at 37 °C with 5 mM hydrogen peroxide (Merck, USA) for 3 h, 6 h and 24 h. Susceptibility of *M. smegmatis* to antibiotics like ethambutol, isoniazid and rifampicin (20 μg/ml) (all from Sigma-Aldrich, USA) and vancomycin (5 μg/ml) and ampicillin (750 μg/ml) (both from Amresco, USA) was determined in 7H9 broth cultured for 24 h and 48 h. Numbers of CFU for each stress and antibiotics were determined by plating serial dilutions onto 7H10 agar plates.

### Mycobacterial lipid extraction and TLC analysis

*M. smegmatis* strains (*Msmeg-pVV* and *Msmeg-PE11*) were grown at 37 °C in 500 ml Middlebrook 7H9 broth supplemented with 0.5% glycerol, 0.05% Tween 80 and 10% OADC until an absorbance of 0.8 at 600 nm was reached. Cells recovered by centrifugation at 5,000 *g* for 10 min and total lipids from the mycobacterial wet pellets (equal weights of both strains) were extracted at room temperature, with 50 ml chloroform-methanol at a ratio of 2:1 (vol/vol). The organic phase containing the whole-cell lipid extracts were evaporated to dryness using rotary evaporator (Heidolph Instruments, India). Lipids were re-suspended in a minimal volume of chloroform and identified by thin layer chromatography (TLC) on Silica Gel 60 plates (Macherey-Nagel, Germany). Polar and apolar lipids were fractionated on a preparative TLC using specific solvent systems. Polar lipids were extracted using chloroform-methanol-water (65:25:4, vol/vol/vol), whereas apolar lipids were extracted using Hexane-ethylacetate (90:10, vol/vol). Glycolipids were extracted from *Msmeg-PE11* or *Msmeg-pVV* using Methanol: Ammonium hydroxide (80:20, vol/vol) as mobile phase and detected by α-naphthol/sulfuric acid staining. The Mycolic acid containing glycolipids were separated from the pool of total polar lipids using Chloroform: Methanol: Ammonium hydroxide (80:20:2 vol/vol/vol) mobile phase and visualized by Iodine vaporization, followed by charring with 5% phosphomolybdic acid.

### Fatty acid composition by gas liquid chromatography

The fatty acid compositions of the lipid fractions were determined by gas liquid chromatography (GLC). The lipid fractions were converted to their corresponding methyl esters and GLC analysis of the fatty acid methyl esters (FAMEs) was performed using a Agilent 6890 gas chromatograph coupled to a flame ionization detector (FID) equipped with a DB 225 capillary column (30 m × 0.25 mm × 0.25 μm; 50% cyanopropylphenyl dimethyl polysiloxane stationary phase material, J & W Scientific, USA), a split injector (split ratio, 10:1), and a FID. The column temperature program was set for 2 min at 160 °C, 5 °C/min to 230 °C and 20 min at 230 °C, respectively. The injector was adjusted to 230 °C with a split ratio of 10:1 using nitrogen carrier gas at a flow rate of 1 ml/min. The detector temperature was 270 °C with air and hydrogen flow rates of 300 ml/min and 30 ml/min, respectively. The fatty acid peaks were identified by comparing the retention times with those of a mixture of standard FAMEs, C4–C24 (Supelco, USA).

### GC/MS studies of fatty acid methyl esters

GC/MS studies were carried out for the identification of FAMEs formed during the transesterification reaction. The GC/MS detection was performed with Agilent 6890 N gas chromatograph connected to Agilent 5973 mass selective detector at 70 eV (*m*/*z* 50–800; source at 230 °C and quadruple at 150 °C) in the EI mode with a HP-1 ms capillary column (30 m × 0.25 mm I.D. × 0.25 μM film thickness). The oven temperature was kept at 100 °C for 2 min and finally raised to 300 °C at 10 °C/min and maintained for 20 min at 300 °C. The carrier gas, helium was used at a flow rate of 1.0 ml/min. The inlet temperature was maintained at 300 °C and split ratio was 50:1. Structural assignments were based on interpretation of mass spectrometric fragmentation and confirmed by comparison of retention times as well as fragmentation pattern of authentic compounds.

### Infection of mice

Six weeks old male Balb/c mice (six per group, housed in the Centre for DNA Fingerprinting and Diagnostics animal facility located within the premises of Vimta Labs, Hyderabad) were infected with 5 × 10^7^ CFUs of either *Msmeg-pVV or Msmeg-PE11* by intravenous injection. All the strains were dispersed in PBS-T and no clumping was observed. The inoculums were cultured on 7H10 plates containing kanamycin and hygromycin B to confirm that equal numbers of bacteria were administered intravenously for each strain. At each time point (day 2, 7 and 14), survival of the recombinant *M. smegmatis* strains in mouse organs was determined using methods as described previously^36^. Briefly, the mice were sacrificed and lungs, liver and spleens were removed aseptically and homogenized in sterile PBS-T. The mouse tissue homogenates were diluted in PBS-T, and 100 μl aliquots from each dilution were plated on Middlebrook 7H10 agar plate containing 10% OADC, 25 μg/ml kanamycin and 50 μg/ml of hygromycin B. The plates were incubated at 37 °C, and the CFUs were determined after 3–4 days. Animal experiments were carried out in accordance with the approved guidelines of the Institutional Animal Ethics Committee (IAEC) of the Vimta Labs Ltd. (IAEC protocol approval number: PCD/CDFD/15).

### Histopathology of infected organs

The histopathology was carried out as described earlier^36^. Briefly, lung, liver and spleen of mice was aseptically removed from euthanized animals and were fixed in 10% formalin and then embedded in paraffin wax. Sections were then stained with hematoxylin and eosin stain for visualizing under an Olympus CX21 microscope (Olympus, Japan). Also, microphotographs were taken using an Olympus DP72 CCD camera, (Japan) attached to the microscope. DP2-BSW software was used for image analysis. A group of 10 mice were used for determining the survival index infected with either *Msmeg-pVV* or *Msmeg-PE11* strain for 30 days post-infection.

### Statistical analysis

Data were expressed as mean ± SD and Student’s t test was used to determine statistical differences between the groups and *p* < 0.05 was considered to be significant. GraphPad Prism 5.02 software (GraphPad software) was used for statistical analysis.

## Additional Information

**How to cite this article**: Singh, P. *et al.* PE11, a PE/PPE family protein of *Mycobacterium tuberculosis* is involved in cell wall remodeling and virulence. *Sci. Rep.* **6**, 21624; doi: 10.1038/srep21624 (2016).

## Supplementary Material

Supplementary Information

## Figures and Tables

**Table 1:** Fatty acid composition of *Msmeg-pVV* and *Msmeg-PE11* estimated by GC, GC/MS analysis.

	Fatty Acid Compositions (Wt %)															
	14:0	15:0	16:0	16:1	17:0	17:1	18:0	18:1	18:0 (10-methyl)	18:2	20:0	20:1	22:0	22:1	24:0	24:1
*Msmeg-pVV* Total	2.6	0.3	18.5	13.8	0.4	1.1	7.4	44.2	6.3	0.8	1.0	0.4	0.9	–	2.3	–
*Msmeg-PE11* Total	3.1	0.3	17.9	13.8	0.3	0.9	6.2	41.5	9.7	0.9	0.8	0.4	1.0	–	3.2	–
*Msmeg-pVV* Polar	2.2	0.5	26.3	10.7	0.5	0.6	10.1	35.4	11.6	1.5	0.2	0.2	0.1	0.1	–	–
*Msmeg- PE11* Polar	2.3	0.5	25.6	9.8	0.5	0.7	7.6	35.9	15.2	0.4	0.2	0.2	0.2	0.9	–	–
*Msmeg-pVV* Apolar	2.8	0.3	15.4	14.9	0.4	1.0	6.5	46.0	3.6	2.6	1.2	0.5	1.2	0.1	3.2	0.3
*Msmeg-PE11* Apolar	2.3	0.2	14.5	13.4	0.3	1.0	5.5	44.7	5.1	2.7	1.3	0.6	1.7	0.2	5.9	0.6

The number CX:Y, for example, indicates the carbon number *X* and *Y* double bond(s).
