# Supplementary material for: PE11, a PE/PPE family protein of Mycobacterium tuberculosis is involved in cell wall remodeling and virulence
Source: Sci Rep. 2016 Feb 23;6:21624. doi: 10.1038/srep21624 (PMC4763214; doi:10.1038/srep21624)
Supplement: Supplementary Information [file srep21624-s1.pdf]

PE11, a PE/PPE family protein of *Mycobacterium tuberculosis* is involved in cell wall remodeling and virulence

Parul Singh[1,2], Rameshwaram Nagender Rao[1], Jala Ram Chandra Reddy[3], R.B.N. Prasad[3], Sandeep Kumar Kotturu[4], Sudip Ghosh[4] and Sangita Mukhopadhyay[1,*]

1 Laboratory of Molecular Cell Biology, Centre for DNA Fingerprinting and Diagnostics (CDFD), Nampally, Hyderabad, India

2 Graduate Studies, Manipal University, Manipal, Karnataka, India

3 Centre for Lipid Research, CSIR-Indian Institute of Chemical Technology, Uppal Road, Hyderabad, India

4 Molecular Biology Division, National Institute of Nutrition (ICMR), Jamai-Osmania PO, Hyderabad, India

***To whom correspondence should be addressed:** Sangita Mukhopadhyay, Laboratory of Molecular Cell Biology, Centre for DNA Fingerprinting and Diagnostics (CDFD), Bldg. 7, Gruhakalpa, 5-4-399/B, Nampally, Hyderabad - 500001, India. Tel: 91-40-24749423; Fax: 91-40-24785447; E-mail: sangita@cdfd.org.in; sangitacdfd@gmail.com

Supplementary Figure 1

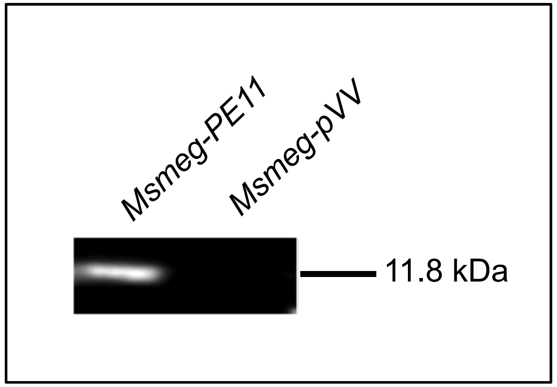

Supplementary Figure 1: Expression of *M. tuberculosis* PE11 in *M. smegmatis* mc^{2}155 bacteria. *M. smegmatis* harboring either the backbone vector (*Msmeg-pVV*) or the *pe11* gene cloned into pVV16 vector (*Msmeg-PE11*) were cultured at 37°C in 7H9 medium with 0.05% Tween 80 and at an OD 0.6. The bacterial cell lysates were loaded (50 µg) onto an SDS-PAGE gel and subjected to immunoblot analysis to detect His tagged PE11 protein using rabbit anti-Histidine tag antibody as primary and HRP conjugated goat anti-rabbit IgG as the secondary antibody. The blot was visualized by chemiluminescence following the manufacturer's instructions (GE Healthcare, UK).

Supplementary Figure 2

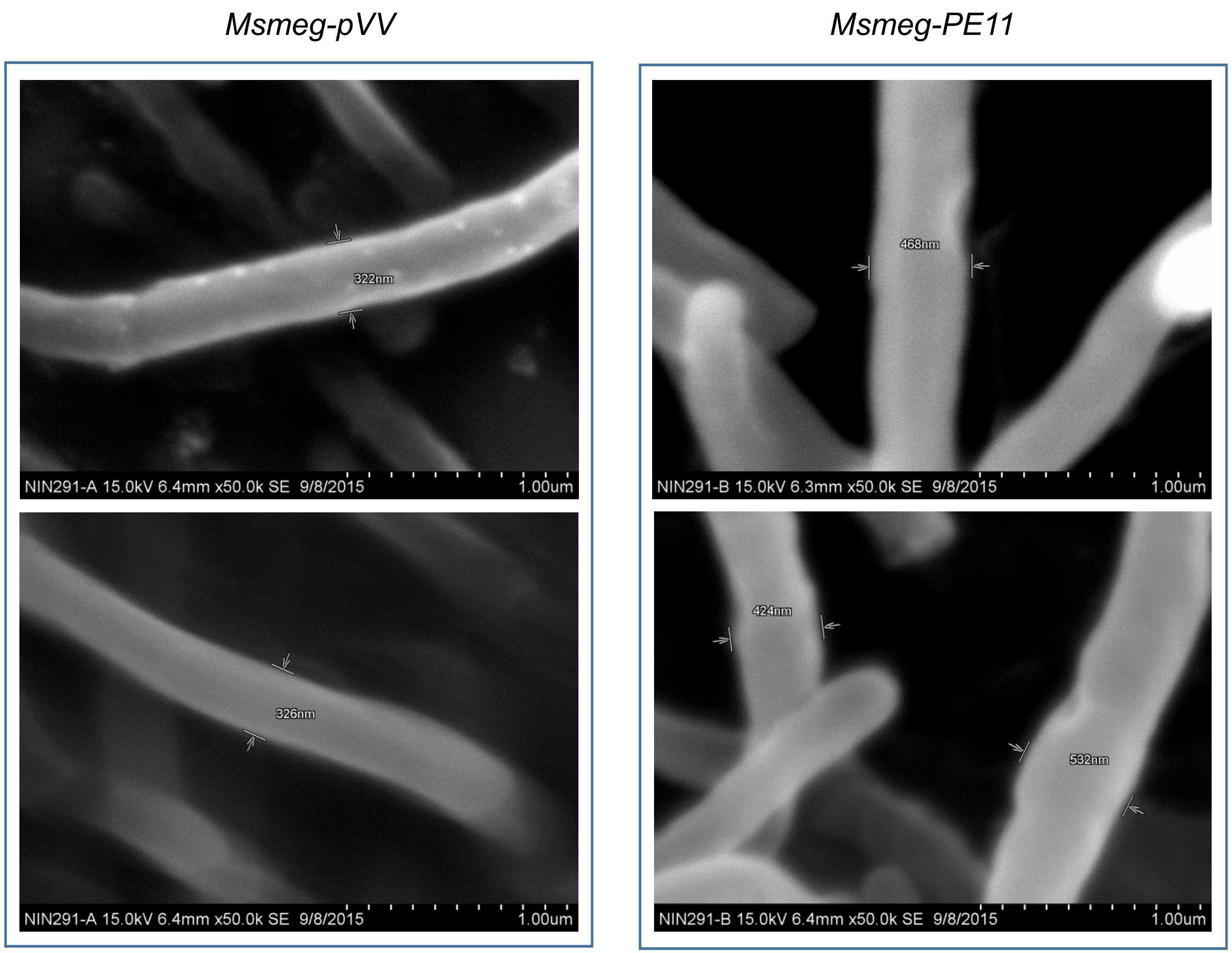

Supplementary Figure 2: PE11 causes an increase in cell width of *M. smegmatis*. *Msmeg-pVV* or *Msmeg-PE11* were cultured in 7H9 medium with 0.05% Tween 80 and at an OD 0.6 ($OD_{600\ nm}$), bacteria were harvested for scanning electron microscopy (SEM) analysis to compare cell width. Scale bar, 1 µm. Two representative pictures for *Msmeg-pVV* (left column) and *Msmeg-PE11* (right column) are shown.

Supplementary Figure 3

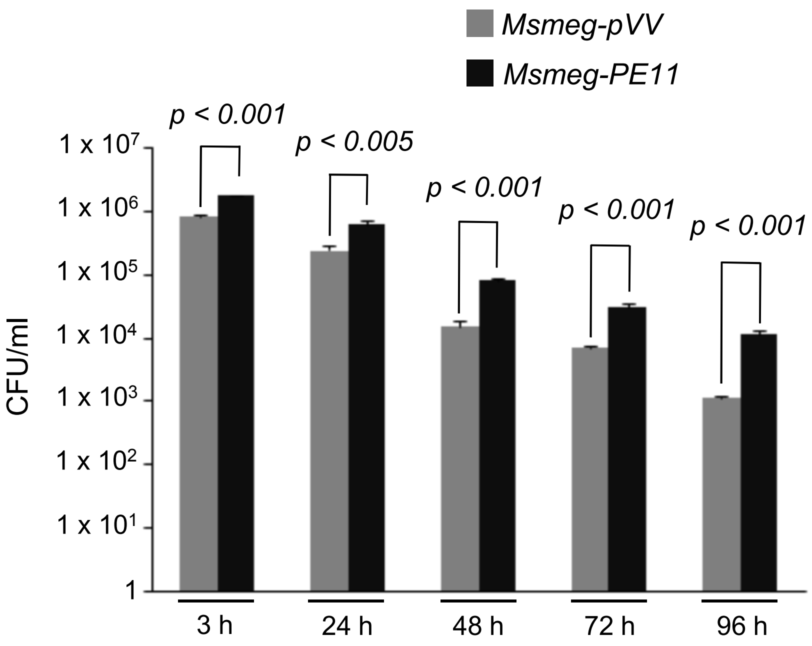

Supplementary Figure 3: Expression of PE11 in *M. smegmatis* enhances intracellular survival of the bacilli in Balb/c peritoneal macrophages. The thioglycollate elicited peritoneal macrophages (0.5×10^6 cells/well/ml in 24 well tissue culture plate) from Balb/c mice were infected with *Msmeg-pVV* or *Msmeg-PE11* at 1:10 MOI and harvested at various time points post-infection. Cells were next washed, lysed and plated in triplicate on Middlebrook 7H10 agar plates supplemented with 10% OADC, 25 µg/ml kanamycin and 50 µg/ml hygromycin B for CFU counts. Results are representative of mean ± SD of three different experiments.

Supplementary Figure 4

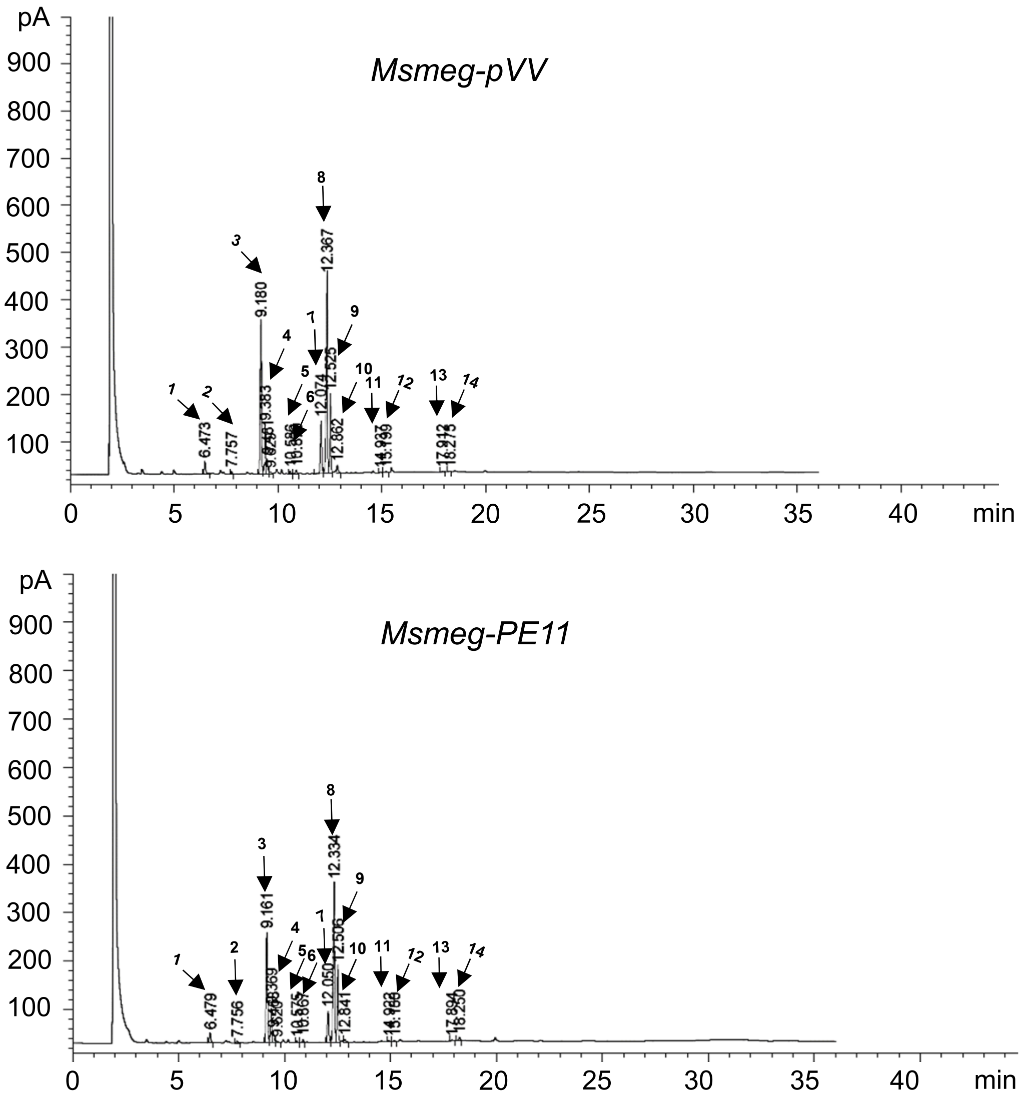

Supplementary Figure 4: Typical GC chromatograms of *Msmeg-pVV* and *Msmeg-PE11*. Peaks of fatty acid methyl esters of *Msmeg-pVV* or *Msmeg-PE11* : 1, C14:0; 2, C15:0; 3, C16:0; 4, C16:1; 5, C17:0; 6, C17:1; 7, C18:0; 8, C18:1; 9, C18:0-10 methyl; 10, C18:2; 11, C20:0; 12, C20:1; 13, C22:0; 14, C22:1; the number of X:Y, e.g., indicates the carbon number X and Y double bond(s).

Supplementary Figure 5

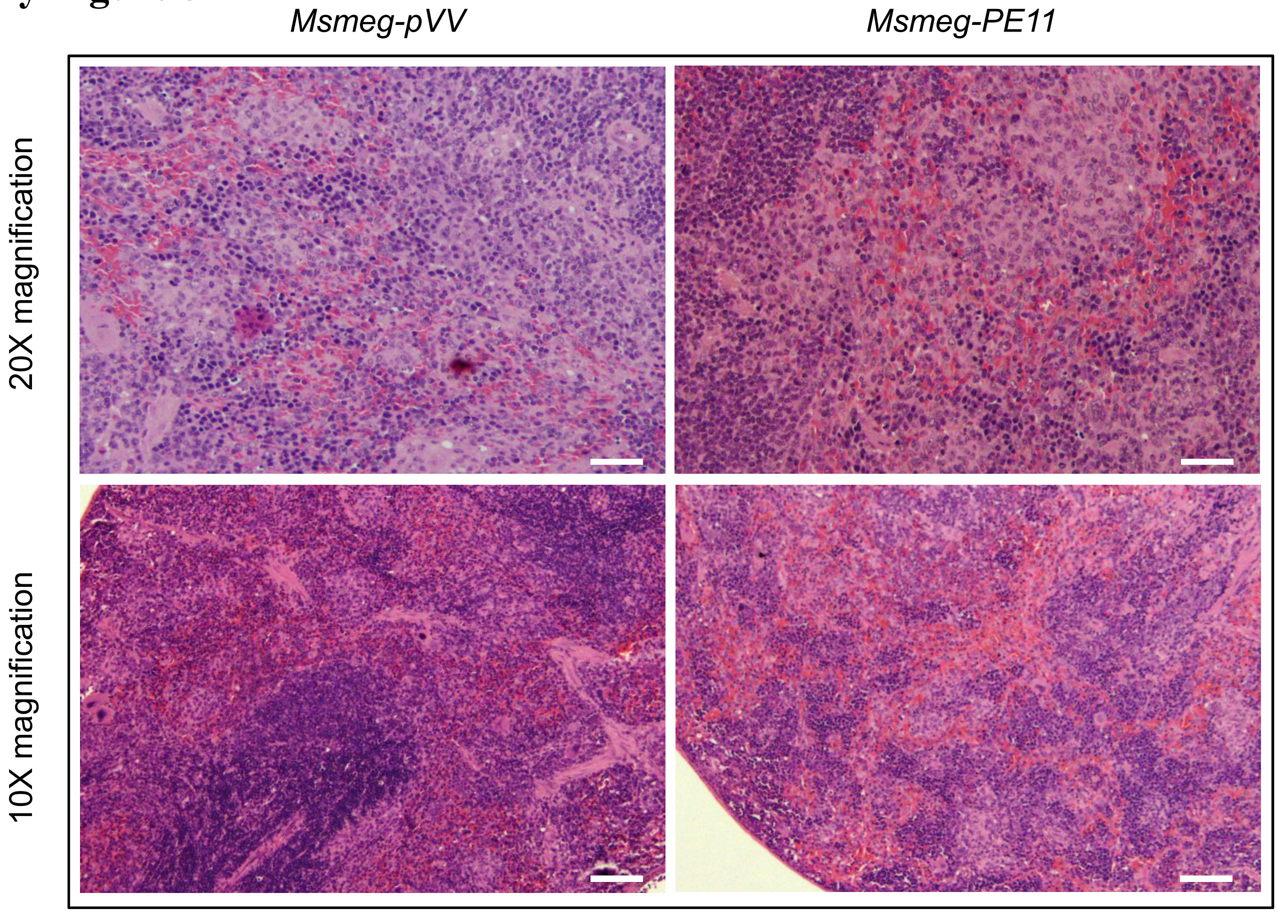

Supplementary Figure 5: Histopathology of spleen of Balb/c mice infected with *Msmeg-pVV* or *Msmeg-PE11* at day 14 post-infection. H & E stained spleen sections from *Msmeg-pVV* or *Msmeg-PE11* strain infected mice were observed at 10X or 20X magnification. Scale bar, 200 µm.

Supplementary Figure 6

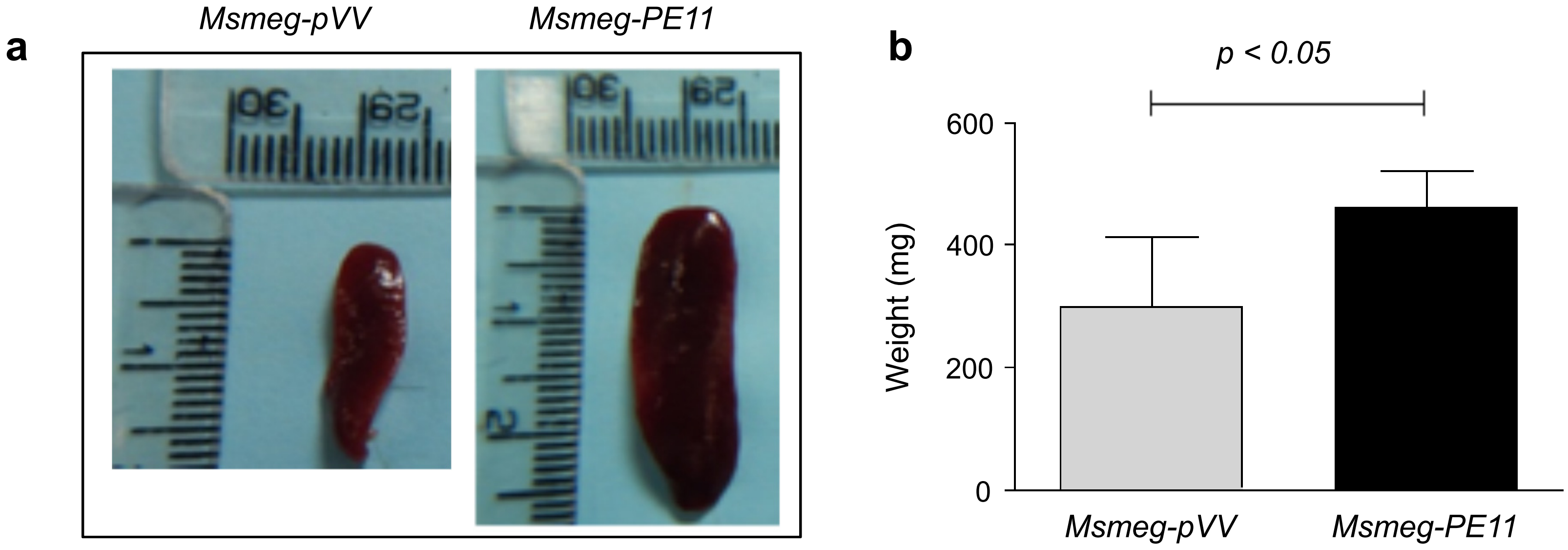

Supplementary Figure 6: Infection of Balb/c mice with *Msmeg-PE11* causes an increase in spleen size and weight. Spleens isolated from Balb/c mice at day 14 after intravenous administration of *Msmeg-pVV* or *Msmeg-PE11* showed an increase in size **(a)** and weight **(b)** when compared with spleen of Balb/c mice infected with *Msmeg- pVV*. Data shown is mean ± SD for 6 mice per group.

Supplementary Figure 7

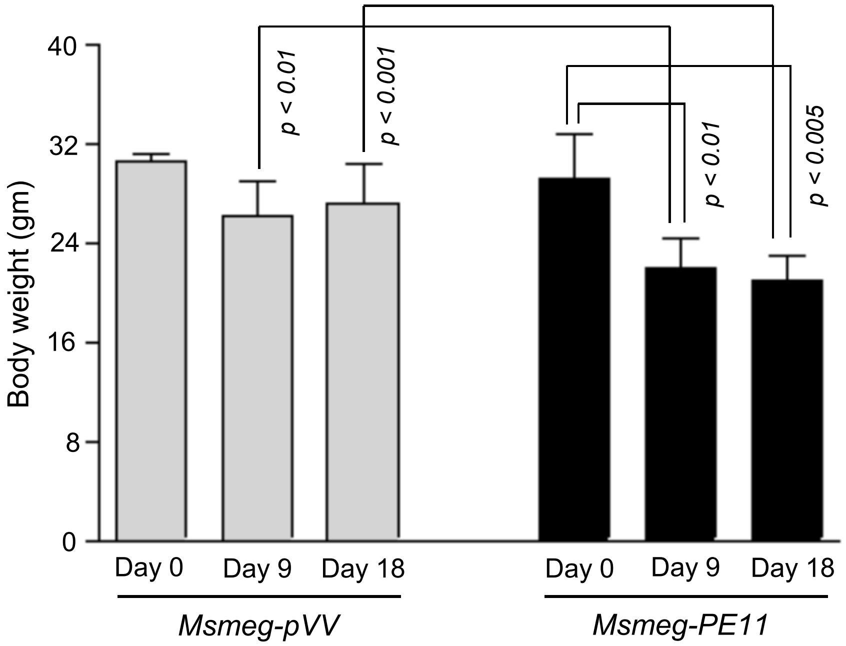

Supplementary Figure 7: Decreased body weight of mice infected with *Msmeg-PE11* compared to *Msmeg-pVV*. Body weight of mice was taken before (day 0) and 9 and 18 days after they were infected with either *Msmeg-pVV* or *Msmeg-PE11*. Data shown are mean ± SD for a total of 7 mice.

Supplementary Figure 8

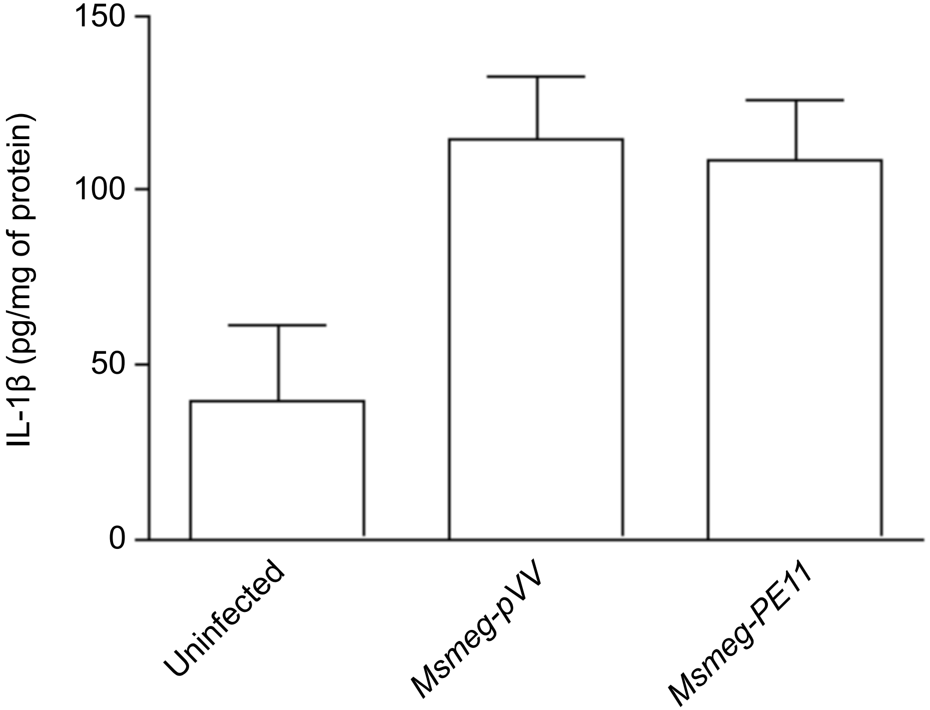

Supplementary Figure 8: Figure. IL-1β profile of Balb/c mice infected with *Msmeg-pVV* and *Msmeg-PE11* at day 7 post-infection. Balb/c mice were infected intravenously with 5 × 10^7 *Msmeg-pVV* or *Msmeg-PE11*. At day 7 post-infection mice were sacrificed and lungs were harvested and homogenized in RIPA buffer containing protease inhibitor. After centrifugation at 14,000 rpm for 20 minutes at 4°C, the supernatants were collected and total protein concentrations in the lung tissue homogenates were determined using a BCA kit (Thermo Scientific, USA). The levels of IL-1β in lung tissue homogenates were measured by two-site sandwich enzyme immune assay (EIA) following the manufacturer's protocol (BD Pharmingen, San Jose, CA, USA). Standard curves for IL-1β was obtained using the recombinant standard protein provided along with the kit. Data shown are mean ± SD of 4 mice per group.

Supplementary Figure 9

Primers	Sequence (5'-3')	Annealing Temperature (°C)
**GAPDH-F**	ACAACTTTGTCAAGCTCATTTCC	59
**GAPDH-R**	GATAGGGCCTCTCTTGCTCA	
**IL-1β-F**	CCTCAATGGACAGAATATCAACC	62.5
**IL-1β-R**	GATCCACACTCTCCAGCTG	
**TNF-α-F**	GCTTTCCGAATTCACTGGAGC	57
**TNF-α-R**	CACCTCAGGGAAGAATCTGGA	
**IL-4-F**	CGGCACAGAGCTATTGATGGG	61.4
**IL-4-R**	CCCTTCTCCTGTGACCTCGT	
**IL-10-F**	CTGAAGACCCTCAGGATGCG	57
**IL-10-R**	GACACCTTGGTCTTGGAGCTTA	

Supplementary Figure 9: Sequence of the primers used for RT-qPCR.
